# New advances in probing cell–extracellular matrix interactions

**DOI:** 10.1039/c6ib00251j

**Published:** 2017-03-27

**Authors:** Allen P. Liu, Ovijit Chaudhuri, Sapun H. Parekh

**Affiliations:** a Department of Mechanical Engineering , University of Michigan , Ann Arbor , MI 48109 , USA . Email: allenliu@umich.edu; b Department of Biomedical Engineering , University of Michigan , Ann Arbor , MI 48109 , USA; c Cellular and Molecular Biology Program , University of Michigan , Ann Arbor , MI 48109 , USA; d Biophysics Program , University of Michigan , Ann Arbor , MI 48109 , USA; e Department of Mechanical Engineering , Stanford University , Stanford , CA 94305 , USA . Email: chaudhuri@stanford.edu; f Department of Molecular Spectroscopy , Max Planck Institute for Polymer Research , Mainz 55128 , Germany . Email: parekh@mpip-mainz.mpg.de

## Abstract

The extracellular matrix (ECM) provides structural and biochemical support to cells within tissues. An emerging body of evidence has established that the ECM plays a key role in cell mechanotransduction – the study of coupling between mechanical inputs and cellular phenotype – through either mediating transmission of forces to the cells, or presenting mechanical cues that guide cellular behaviors. Recent progress in cell mechanotransduction research has been facilitated by advances of experimental tools, particularly microtechnologies, engineered biomaterials, and imaging and analytical methods. Microtechnologies have enabled the design and fabrication of controlled physical microenvironments for the study and measurement of cell–ECM interactions. Advances in engineered biomaterials have allowed researchers to develop synthetic ECMs that mimic tissue microenvironments and investigate the impact of altered physicochemical properties on various cellular processes. Finally, advanced imaging and spectroscopy techniques have facilitated the visualization of the complex interaction between cells and ECM *in vitro* and in living tissues. This review will highlight the application of recent innovations in these areas to probing cell–ECM interactions. We believe cross-disciplinary approaches, combining aspects of the different technologies reviewed here, will inspire innovative ideas to further elucidate the secrets of ECM-mediated cell control.

## Abstract 2

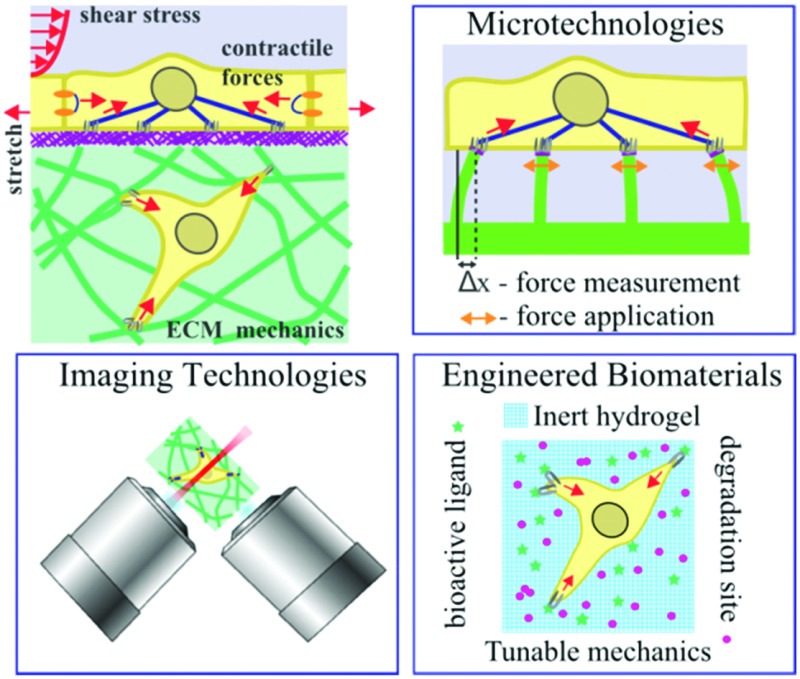
This review highlights the application of recent innovations in microtechnologies, biomaterials, and imaging tools for probing cell–ECM interactions.

Insight, innovation, integration

Recent progress in cell mechanotransduction research – the study of coupling between mechanical inputs and multiscale cell phenotype – has been facilitated by advances of experimental tools, particularly microtechnologies, engineered biomaterials, and imaging and analytical methods. This review will highlight the application of recent innovations in these areas to probing cell–ECM interactions in the context of mechanotransduction. We believe these cross-disciplinary approaches will inspire innovative ideas to further elucidate the secrets of ECM-mediated cell control.

## Introduction

Many of the secrets to life lie outside the cell. The extracellular matrix (ECM), consisting largely of protein biopolymers, provides structural and biochemical support to the cells within a tissue. While the ECM has long been viewed as a static “home” for cells, a growing body of work is revealing that physicochemical properties, such as the stiffness and structure, of ECM can drastically affect cell behaviors in ways similar to soluble biochemical signals.^1^–^4^ In this context, interactions with the ECM regulate signaling and gene expression that underlie cellular processes during development,^5^,^6^ homeostasis,^7^,^8^ wound healing,^9^ and cancer invasion.^10^ Research in the emerging field of cell mechanotransduction is beginning to unravel the complex connections between cells sensing the physicochemical properties of the ECM and modulation of intracellular signaling.

**Fig. 1:**
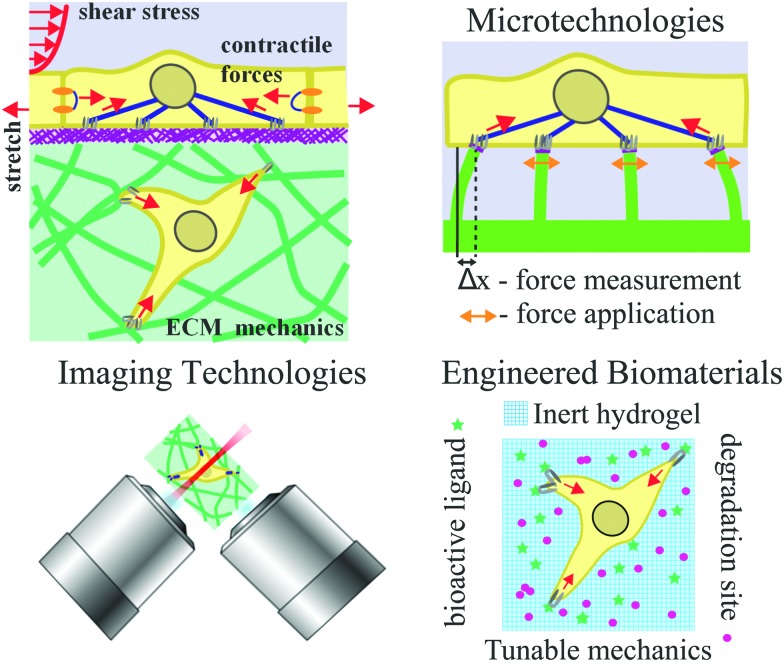
Overview of cell–ECM interactions (top left) and thematic topics covered in this review: microtechnologies (top right), engineered biomaterials (bottom right), and imaging technologies (bottom left). Forces are indicated by red arrows.

The ECM in the cell's microenvironment presents a set of passive mechanical properties that regulate a range of cellular behaviors (Fig. 1). Externally applied, or active, mechanical input can also manifest *via* cell–ECM interaction to influence mechanical properties of cells or elicit biological responses; passive and active inputs are described in more detail in the next section. Conventional cell biology tools do not provide a means to manipulate the physical, geometrical, and mechanical aspects of cells’ microenvironment. Since a cell's size is ∼10–100 μm, specialized approaches need to be developed to exert and detect forces on the length scale of single cells for studies of mechanotransduction. Microtechnologies, developed by engineers, chemists, and physicists, have made a significant impact in our abilities to control passive and active mechanical inputs.

In addition to measuring and exerting forces on cells, the so-called passive microenvironment – defined as the chemical and mechanical nature of the ECM supporting the cell – is crucial for determining cell behavior and cell fate. The importance of the ECM is exemplified by the fact that modifying only the ECM can profoundly influence stem cell differentiation^11^ or the malignant phenotype of mammary epithelial cells.^12^ When considering these findings in the context of the large variation of mechanical and morphological properties of body tissues, it is not surprising that the nature of the ECM strongly influences cell fate. Indeed, the increasing number of studies demonstrating a comparable, if not larger, role that the ECM properties play in dictating cell behavior compared to soluble cues has led to an explosion of ECM-mimicking biomaterials. These materials range from being completely natural, such as collagen gels, to fully synthetic, such as synthetic poly(ethylene glycol) hydrogels, with varying morphological and mechanical properties. Numerous examples and general paradigms learned regarding the ability of engineered ECMs to control cell fate are discussed in this review.

While developments in microtechnologies and engineered biomaterials are unquestionably important to studies of cell–ECM interaction, advances in high-resolution imaging and analytical technologies have provided methods to visualize and quantify this interaction with unprecedented precision. Specifically, improvements in high-resolution three-dimensional (3D) fluorescence imaging, correlative electron microscopy and super-resolution imaging, and label-free microscopy techniques have permitted quantification of structural and morphological changes in cell–ECM systems from the molecular to macro-scale level. For example, visualizing specific protein localization in focal adhesion plaques,^13^ ultrastructural changes in chromatin structure resulting from changes in ECM mechanics,^14^ or 3D cytoskeletal reorganization in response to different ECM mechanics^15^ are examples of phenotypic responses that have been observed using advanced imaging technologies.

Integration of cellular micromanipulation with custom-designed biomaterials and advanced imaging and analytical methods comprises a multifaceted toolbox to answer fundamental questions about the nature of cell–ECM interactions and the reciprocal relationship between cells and their ECM. As these separate communities continue to advance, it will be imperative to continue pushing newer technologies in these, and other, fields together in order to predict, and eventually control, how active mechanical inputs to cells and passive inputs from the surrounding ECM synergize to dictate cell fate in pathogenesis and development. In the sections that follow we give an overview of mechanotransduction, followed by a review of how advances in microtechnologies, engineered biomaterials, and imaging and analytical methods have contributed to our understanding of cell–ECM interactions in different physiological and cellular contexts.

## Overview of mechanotransduction

### Physiological relevance of cell–ECM mechanotransduction

Mechanotransduction is known to play a key role in many physiological processes in development, homeostasis, regeneration, aging, and disease.^16^ There are two main modalities of mechanotransduction. Tissues and cells can respond directly to mechanical loading, which we describe as an active mode of mechanotransduction. Alternatively, cells can sense and respond to alterations in mechanical properties of their surroundings, which we describe as a passive mode of mechanotransduction. In this section, we describe some selected examples of these two modes of mechanotransduction and discuss the role of the ECM in each mode. The reader is referred to other excellent reviews and books for a more thorough description of the physiological context for mechanotransduction.^17^–^20^

In the active mode of mechanotransduction, tissues and cells directly sense and respond to mechanical loading. One example of this is in mechanosensory cells or neurons that directly convert mechanical loading to a biochemical signal and underlie our sense of touch as well as hearing.^21^,^22^ Auditory hair cells convert differential lateral motion between the tectorial membrane, attached to the apical surface of the cell, and the basilar membrane into electrical signals, while Merkel cells embedded in the epidermis are thought to respond to mechanical force and deformation and mediate touch sensation. The skeletal system serves as another important example as it is exquisitely responsive to mechanical loading.^17^ For example, loading is an important driver in maintenance of bone mass and architecture. Indeed, it is commonly known that astronauts or bed-ridden patients rapidly lose bone mass. Beyond playing a role in homeostasis, mechanical loading also plays a key role in development of the skeletal system, as formation of articular cartilage, fibrocartilage, fibrous tissue, and bone are linked closely to mechanical stress history. For example, articular cartilage is associated with a history of compressive hydrostatic stress while fibrous tissue is associated with a history of shear stress or tension.^23^ Many tissues generate large contractile forces, including heart muscle, skeletal muscle, the gastrointestinal tract, or lung, and these forces have been found to provide an important biological cue for development, maintenance, and function of these tissues. Initiation of heart beats drive development of the vasculature and heart development.^24^ The onset of atherosclerosis has been linked to disturbed or turbulent shear flow, and therefore altered shear stress on the vascular endothelial cells.^25^,^26^ The commonly observed generation of increased skeletal muscle mass due to weight lifting demonstrates the phenomena of skeletal muscle adaptation.^27^ Associated growth of skin to cover the increased muscle mass, or adipose tissue in other contexts, are examples of how skin grows in response to sufficient stretch.^28^ When skin is wounded, tension across the wound site can cause hypertrophic scar formation.^29^ In lungs, fetal breathing, and the resulting compression and tensile forces, regulates lung development and deep breaths stimulate surfactant secretion by airway epithelial cells.^30^

As tissues consist of cells and ECM, the ECM plays a pivotal role in mediating the response to mechanical forces. In some cases, such as touch sensation by Merkel cells,^31^ the ECM can play a role in mediating the transmission of force. In other cases, the ECM provides anchorage for cells to respond to mechanical cues. For example, endothelial cells, which sense and respond to shear stress provided by blood flow on their apical surfaces, are mechanically anchored to a basement membrane matrix on their basal surfaces, and this anchorage is necessary for the cells to respond normally to shear stresses.^25^,^26^

In the passive mode of mechanotransduction, cells sense and respond to mechanical properties of the ECM as opposed to external loading. Awareness of this mode of mechanotransduction has emerged from its suspected role in various pathologies, whose pathogenesis is associated with alterations in mechanical properties. There are numerous examples of such pathologies. For example, tumors are stiffer than normal tissue and breast cancer progression is associated with progressive stiffening of breast tissues.^10^ In diseases of the lung, emphysema is marked by a reduction in lung elasticity,^32^ while pulmonary fibrosis is associated with stiffening of lung tissues.^33^ More generally, the elastic moduli of body tissues range from as low as ∼100 Pa in adipose, breast, and brain tissues and up to hundreds of MPa in tendon, and even GPa in bone.^20^ In each of these tissues, adherent cells exert traction forces on substrates at the sites of adhesions,^34^ which means that cell–ECM interactions are likely to be continuously mediated by tissue mechanics. Indeed, an emerging body of evidence has accumulated that provides strong support for this idea and substantial work has now shown that ECM mechanics plays a potent role in directing cell behavior in various contexts, including cell migration and cell differentiation.^1^,^2^ The ability to control cell behavior with changes in ECM mechanics demonstrates the specificity of the cell–ECM interaction for different *in vivo* mechanical environments. Some of these results are described in this review.

### Current understanding of mechanotransduction

While both passive and active mechanical inputs are known to play important roles in physiology and pathology, the molecular basis of mechanotransduction at the cellular and subcellular levels remain relatively unclear. In both passive and active modes of mechanotransduction, it is generally accepted that mechanical signals from the ECM are sensed through focal adhesion assemblies and transduced *via* the actin cytoskeleton networks. In the passive mode of mechanotransduction, a conceptual picture has emerged in which cells exert traction forces on the ECM, in a process mediated by formation of focal adhesions, Rho activation, and actomyosin contractility, and cells subsequently gauge the resistance to the traction forces provided by the substrate.^1^,^12^,^35^–^37^ Important studies have revealed that focal adhesion proteins talin and vinculin undergo conformational changes upon tensile force application both *in vitro*^38^ and in cells.^39^ Nuclear lamin-A concentration responds to substrate stiffness and regulates the cellular response to stiffness, possibly through its role in nuclear stiffness and mechanical coupling between the nucleus and actin cytoskeleton.^40^ Furthermore, recent work has highlighted a link between the YAP/TAZ transcription factor localization and intracellular tension by establishing that YAP/TAZ activation mediates the response to ECM stiffness and adhesion geometry with respect to apoptosis, proliferation, and differentiation of mesenchymal stem cells (MSCs).^41^ Together these studies show the direct impact of mechanical inputs on cytoskeletal protein organization and genetic regulation, respectively. Reinforcing these findings, very recent work has identified another transcription factor relocalizing between the nucleus and cytoplasm in response to ECM mechanical stiffness^42^ and additional cytoskeletal elements that impact cell phenotypes, specifically gene expression, with respect to changes in ECM mechanics.^43^ In the active mode of mechanotransduction, various mechanisms have been implicated, including mechanically gated ion channels that open up directly in response to force,^44^ direct transmission of force to the nucleus through the cytoskeleton,^45^,^46^ and conformational changes, strain, or unfolding of proteins under force that alter biochemical activities.^47^–^49^ Though some general mechanisms underlying mechanotransduction have been established, whether, and to what extent, additional avenues for “relaying” mechanical signals to regulate short or long timescale cell behavior is still very much unknown and is an active area of study.

### Measuring forces at the cell–ECM interface

Measuring forces at the cell–ECM interface is a critical aspect of fully understanding cell–ECM interactions. A number of approaches have been developed over the years for measuring cellular forces. The most commonly used method is traction force microscopy (TFM) where fluorescent microparticles embedded in a hydrogel are used for measuring cell-generated traction stresses, by comparing bead positions before and after cells are removed. This technique has mostly been used in planar 2D systems,^50^–^53^ but can also be applied for 3D traction measurements.^54^ Although TFM has been adopted by many groups, the measurement requires microparticle tracking and a mechanical model for the ECM, and TFM can be computationally expensive for 3D environments. Another microscopy-based technique that has been actively used in recent years is Förster resonance energy transfer (FRET)-based biosensors. FRET biosensors have traditionally been used for monitoring cell signaling activities in real time and is based on the idea that the distance between a donor and an acceptor fluorescence moiety will change upon protein activation.^55^ FRET has also been used to demonstrate that cell-generated forces can mechanically unfold fibronectin.^56^ By sandwiching donor and acceptor fluorescence proteins between vinculin head and tail domains connected by an elastic linker, Grashoff, Hoffman, Schwartz and co-workers developed the first tension sensor for measuring forces within single focal adhesions.^57^ By tethering such FRET sensors to ECM binding motifs, the distribution of forces generated by individual integrins can be visualized.^58^ More recently, DNA-based probes have been used as force sensor for cell–ECM interactions. A tension gauge tether was developed based on the use of DNA duplex helix that has a tunable tension tolerance and was used to show the force requirement to activate Notch receptors.^59^ Using DNA hairpins that unfold in response to precise amounts of force, a few groups have measured traction stresses exerted by cells.^60^,^61^ Along with increasing use of optical microscopy-based force reporters for cell–ECM research, there is also the exciting development in optogenetic control of cellular forces by using light-gated dimerization system that has been used for subcellular activation of RhoA.^62^ For a more comprehensive survey on the available optical tools for measuring cell-generated forces, readers are referred to two recent excellent review articles.^63^,^64^

## Microtechnologies in research on cell–ECM interaction

**Fig. 2:**
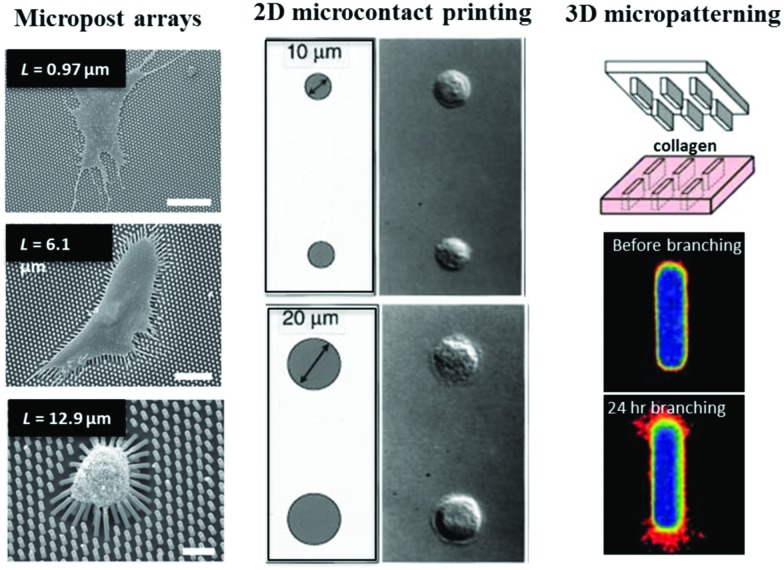
Key developments in microtechnologies for studying cell–ECM interaction. (left) PDMS micropost arrays were used to study how substrate stiffness regulates cell contractility. Scale bars are 50, 30, and 10 μm, respectively from top to bottom. Panel reprinted by permission from Macmillan Publishers Ltd: Nature Methods, ref. 69, copyright 2010. (middle) Cell spreading can be controlled by microcontact printing of ECM proteins. Panel from ref. 66. Reprinted with permission from AAAS. (right) 3D micropatterning of collagen gels enabled the study of how geometry determines site of mammary branching morphogenesis. Panel from ref. 4. Reprinted with permission from AAAS.

The development of microtechnologies for cell–ECM interaction is founded on the interest to create biomimetic cellular microenvironments, which can be traced back to the recognition of two important features/roles of ECM: (1) ECM organization defines tissue stiffness and (2) the ECM presents a complex adhesive surface. Two pioneering works in the late 1990's have inspired the use of microtechnologies to define passive mechanical inputs for single cell mechanotransduction research. Using polyacrylamide gels that are commonly used in gel electrophoresis, Pelham and Wang developed a polyacrylamide-based, collagen-coated flexible substrate with different stiffness by varying gel cross-linking density.^65^ As a result of this development, the study was the first to demonstrate that cells respond to differences in substrate stiffness by altering focal adhesion structures and cell motility. Around the same time, Chen, Ingber and co-workers used micropatterned substrates to define 2D regions of ECM-coated adhesive surface and discovered that cell growth or death was governed by cell shape.^66^ This intriguing finding, and the implication of this work in developmental regulation, highlighted the importance of geometry in control of cellular processes. Both of these early studies motivated new microtechnology development and application and initiated a range of fundamental studies that have significantly contributed to our growing understanding of single cell mechanotransduction (Fig. 2).

### Microfabricated posts for controlling substrate stiffness

While the preparation of polyacrylamide gels with different stiffness is straightforward, an alternative approach to independently control mechanical properties and surface chemistry was developed. Microfabricated post-array-detectors (mPADs),^67^ also sometimes referred to as posts or pillars, is based on the application of soft lithography in printing and molding from elastomeric stamps.^68^ mPADs are made with polydimethylsiloxane (PDMS) and present an array of vertical posts whose ECM-coated tips all lie in one plane. The height, post diameter, and center-to-center distance between posts can all be precisely controlled. Two features afforded by mPADs are particularly attractive to single cell mechanotransduction studies. The slender posts serve as force sensors where the deflection of a post is directly proportional to the force produced (for small deflections). By changing the post geometry, post stiffness can be varied without changing the bulk material properties or surface chemistry. Short posts are more difficult to deflect and thus have higher stiffness compared to taller posts. Inherent to the microfabrication approach is the high spatial regularity of the posts that makes analysis easier, and it was revealed that contractile forces increase with cell spreading^67^ (Fig. 2, left panel).

mPADs were a major development that has clarified important temporal relationships between focal adhesion size, force, and stress^69^ and the origin of mechanical homeostasis^70^ that would otherwise not be possible to resolve using other approaches. Focal adhesion area and traction force both scale with increasing substrate stiffness, and this leads to cell migration towards stiffer substrate driven by cytoskeleton polarization.^71^ mPADs can also be used for force measurement of single contracting cells, and it has been shown that the adhesion strength of laminin to microposts directly influences contractility of single neonatal cardiomyocytes that span across two adjacent microposts.^72^ Micropost arrays have also been utilized for studying collective 3D migration from explanted embryonic tissue from *Xenopus laevis*,^73^ with the microposts serving as physical barriers that modify the topography of the cell–ECM interaction. mPADs can be combined with microcontact printing (discussed further below), and this combination was used to realize that cell shape and substrate stiffness both regulate cell stiffness.^74^,^75^ The same platform has also enabled measurement of contractile forces in different cell types.^76^–^78^ More recently, using sub-micron diameter microposts, it was discovered that actomyosin-based contractile units resembling muscle sarcomere are responsible for stiffness sensing,^79^ demonstrating local stiffness sensing instead of the large-scale (whole cell) mechanosensing previously thought to govern stiffness sensing.^71^ For additional information on the various usages of mPADs, readers are referred to other excellent reviews.^80^,^81^

### Microcontact printing for controlling cell size and shape

From the seminal work showing cellular responses can be geometrically controlled by the extent of cell–ECM interaction, microcontact printing has been used by many groups to investigate how cell adhesion to 2D ECM surfaces regulates different cellular processes. Microcontact printing uses a flexible PDMS stamp with a desirable relief pattern to define sizes and shapes of islands where ECM proteins are stamped (Fig. 2, middle panel), which can range from subcellular to cellular to multicellular sizes. ECM proteins such as collagen, laminin, or fibronectin can be easily deposited to the patterned substrate directly or *via* self-assembled monolayers of alkanethiols on gold with the desirable patterns. By chemically creating non-adhesive areas, cell attachments are restricted to the ECM-patterned areas. Cell-sized micropatterns confine single cells and studies have investigated how cell spreading size or cell tension influence cell motility,^82^–^84^ cytoskeleton organization,^85^,^86^ focal adhesion assembly and cell–cell adhesion,^87^–^89^ cell contractility and traction stress,^50^,^90^,^91^ cell division,^92^ cell differentiation,^93^,^94^ clathrin-mediated endocytosis,^95^,^96^ and macrophage and platelet functions.^97^,^98^ Increasing cell adhesive areas increases focal adhesion assembly and strengths up to a threshold area in fibroblasts,^87^ and this presumably has a direct or indirect influence on cytoskeleton/organelle organization and dynamics, cell migration, and membrane trafficking. Interestingly, using microcontact printing of circular patterns, it was discovered recently that a radially symmetrical cell can break symmetry spontaneously and self-organize a chiral pattern of actomyosin network.^99^ Asymmetric patterns can be used to create a range of interfacial geometries at the perimeter between adhesive and non-adhesive areas, and it has been shown recently by Kilian and co-workers that curvature and perimeter geometry govern spatial expression of cancer stem cell markers.^100^

While cell-sized micropatterns are ideal for defining cell spreading, properly spaced micron-sized patterns can organize subcellular focal adhesions.^95^,^101^ The extent of cell spreading correlates with ECM coverage with optimal spreading at above 15% ECM coverage with spacing less than 5 μm (otherwise cells adapt to ECM pattern shape).^101^ Using subcellular micropatterns, it was shown that supermature focal adhesions (8–30 μm long) sustain more stress which in turns permits alpha-smooth muscle actin to be recruited to stress fibers under high tension.^102^ By creating similarly sized focal adhesions with subcellular-scale microcontact printing and measuring traction stresses, Gardel and co-workers showed a strong correlation between focal adhesion size and traction stress during early stages of focal adhesion growth but not for mature focal adhesions.^103^ The proximity to cell edge has a stronger influence on traction stress for mature focal adhesions, highlighting the dynamic and complex force-dependent nature of cell–ECM interaction. Because of the limits in making small-scale patterns, more sophisticated techniques and other patterning approaches have been used to interrogate cell–ECM interactions.

A modified microcontact printing method based on stamp-off can be used to pattern multiple ECM proteins to segregate integrins on the cell surface to investigate how different integrins function cooperate to guide cell migration.^104^ With microcontact printing methods limited to ∼1 μm features, other microtechnologies have afforded true nanoscale ECM patterning. Using a modified subtractive contact printing to immobilize fibronectin to defined nanopatterns, it was revealed that nanoscale adhesive geometry modulates adhesive force,^105^ consistent with an earlier study.^87^ Nanoscale patterns can also be achieved with other non-contact printing method such as dip-pen nanolithography to directly write ECM proteins,^106^ though the process is highly serial and therefore time-consuming. RGD functionalized gold nanoparticles with interparticle spacing ranging from 30 nm to 120 nm can be generated using block copolymer nanolithography.^107^ It was shown that intermediate spacing supports focal contact formation in melanoma cells, but not for 30 or 120 nm spacing. More advanced block copolymer micellar lithography can generate binary nanostructured hydrogels decorated with gold and titanium oxide nanoparticles for orthogonal functionalization with different cell adhesive peptides.^108^

Microcontact printing can also be used to study collective cell behaviors with pattern sizes of tens or hundreds of microns. These large patterns have been used as a model system of epithelial to mesenchymal transition (EMT) and collective behaviors of 2D epithelial monolayers.^109^–^111^ It has been shown recently that cell spreading and intercellular contacts control TGFβ1-induced EMT.^112^ Microcontact printing can also be used in creative ways to allow more complex control of cell–ECM interactions. For instance, damage-free gaps of both convex and concave geometries can be created by using PDMS stencils, and it was shown that the mechanical coupling between actomyosin cable contraction and cell crawling control the dynamics of gap closure.^113^ Using a robotic microcontact printing approach, multiple ECM proteins with different geometries can be patterned to create complex cell culture environments.^114^ Finally, PDMS stamps can be used to mold collagen-enclosed cavities to simulate 3D micro-tissues (Fig. 2, right panel). This has led to the general understanding that sites of local minimum autocrine inhibitory morphogens and high mechanical stress defined by tissue geometry can influence sites of branching morphogenesis.^4^,^115^

### Microfluidic confinement for modeling 3D cell migration

Cell migration is a key process in various stages of development, homeostasis, wound healing, and pathologies that is heavily influenced by cell–ECM interactions. Cell migration through 3D ECM environments can be modeled in a variety of microfluidic devices.^116^,^117^ One important example of this is in the area of cancer, where microfluidic approaches have been used to better understand cell migration during metastasis. During cancer progression, tumor cell invasion requires migration through confined spaces within the 3D tissues, arising both from the tight spaces between endothelial cell barriers as well as within a dense ECM network,^10^,^118^ leading to the natural questions of how cell–ECM interaction is regulated by 3D confinement. In recent years, there have been a number of intriguing studies that use microfluidic channels with various cross-sectional dimensions to recapitulate 3D confined migration. While migration speed was found to decrease with decreasing channel widths,^119^ it was shown that breast cancer cell migration in confined channels did not depend on actin polymerization or myosin II-dependent contractility as in 2D planar migration.^120^ Instead, Konstantopoulos, Sun, and coworkers demonstrated that directed water permeation serves as a major mechanism for confined 3D migration. This ‘osmotic engine model’ works by having polarized distributions of Na^+^/H^+^ pumps and aquaporins that together creates a net inflow and outflow of water at the leading and trailing edges, respectively. Other studies have found that cell migration speed in confined microchannels can be regulated by cortical tension and cortex-surface interaction in such ways that lead to ‘non-wetting’ shape of a cell and facilitate the transition from slow mesenchymal to fast ameboid migration mode.^121^,^122^ Migration in a 3D microenvironment becomes even more interesting when one considers nuclear deformation through confined spaces as being the rate-limiting step for 3D migration.^123^,^124^ Two recent works provide evidence of nuclear envelope rupture during migration in confined microenvironments.^125^,^126^ These studies also point to the previous unknown and critical roles of endosomal sorting complexes required for transport (ESCRT) machinery for nuclear envelope repair in otherwise uncontrolled exchanges of nuclear and cytoplasmic proteins. Intrinsic differences in single cell migration in geometrically confined spaces in microfluidic channels can be harnessed for separating different cancer cell subpopulations.^127^ Together, microfluidic confinement provides a rich avenue for examining some unique aspects of cell–ECM interaction distinct from microcontact printing and micropost arrays.

### Other microtechnology tools for controlling cell–ECM interactions

Microposts and microcontact printing have mostly been used to dissect 2D cell–ECM interactions. Physical factors such as nanotopography of ECM have also been investigated using nanoimprinted polymethylmethacrylate (PMMA)-coated silicon molds^128^ or photolithography followed by reactive ion etching.^129^ In these cases, nanotopography substrates can govern focal adhesion assembly, cell spreading, and cytoskeleton organization. With the increasing interests in mimicking 3D ECM microenvironment (see Engineered biomaterials section below), there are also microtechnology-based efforts to develop robust and high throughput approaches to generate 3D spheroids. In a simple way, a 384-well format microplate can be used for hanging drop culture^130^ and CO_2_ laser ablation of conventional untreated culture dish can also be used to create concave microwells to support growth of multicellular aggregates.^131^

### Development of active control of cell–ECM interaction

Cells not only sense passive inputs, they also have active mechanical responses. These have largely been studied using a variety of single cell manipulation techniques like optical tweezers,^132^ magnetic tweezers,^133^ atomic force microscopy,^134^ and others that are summarized in an excellent review article.^135^ Our survey of microtechnology approaches above has focused on passive mechanical inputs, but microtechnologies have also been applied in order to provide insight into the cellular response to active mechanical inputs. Shear stress by fluid flow simulating the blood-endothelial cell environment or interstitial flow experienced by fibroblasts can be recapitulated in flow chambers^136^,^137^ while cell stretching to induce strain can be achieved using vacuum driven stretching or direct stretching of an elastomeric membrane.^76^,^138^,^139^ Interesting ways to provide active mechanical input have been developed for single cells. By incorporating magnetic beads or nanowires in a polymeric matrix during the microfabrication process, synchronized or isolated deflection of microposts can be induced under a magnetic field gradient.^140^ Independent cyclic stretching of flexible membranes in 24 stretching chambers can be accomplished using the computer-controlled, piezoelectrically actuated pins of a Braille display.^141^ By integrating piezoresistive sensors and piezoelectric actuators onto cantilevers, it is possible to apply very fast mechanical stimuli (<10 μs rise time) to living cochlear hair cells.^142^ Microfluidic tools can enable single cell mechanical actuation to emulate active mechanical stimuli that cells experience in their natural microenvironment. Both cell aspiration to increase cell tension and mechanical compression to single cells are possible with microfluidic devices.^143^,^144^ Finally, recent microfluidic systems aim to recapitulate more physiological conditions have been developed by creating more complex co-culture models that uses vacuum to produce cyclic stretching to imitate lung expansion^145^ or using optically excitable ion channels to simulate neuromuscular junctions.^146^ Microfluidic systems with precise control have made an impact on cell–ECM research in recent years and readers are referred to more comprehensive, excellent overviews of microfluidic platforms for mechanobiology research and 3D culture systems.^117^,^147^ Table 1 summarizes the salient microtechnologies for cell–ECM study described in this section.

**Table 1:** Summary of key microtechnologies for studying cell–ECM interactions

	Microfabricated post	Microcontact printing	Microfluidics
Features	• Controlled post diameter and spacing • Controlled stiffness	• Controlled cell size and shape • Cellular and subcelluar patterns • Works for 3D • Combined with different substrate stiffness	• Active mechanical stimuli (shear flow, aspiration, compression) • 3D confinement for cell–ECM interaction

Limitations	• Constrained cell adhesions • Cannot extend to 3D	• Constrained cell adhesions	• Limited range of substrate stiffness • Difficult to pattern ECM

Applications	• Relationships between focal adhesion size, force, and stress	• Focal adhesion organization • Cell spreading • Single or collective cell migration • 3D microtissues	• Apply defined mechanical stimuli • Confined cell migration • Organ on a chip

## New advances in engineered biomaterials

**Fig. 3:**
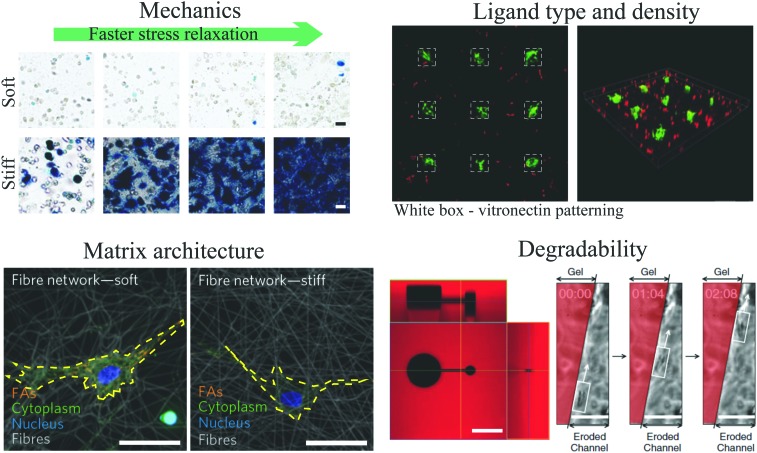
Key parameters of engineered biomaterials for 3D culture. (top left) Differentiation of MSCs in RGD-coupled alginate hydrogels depends on both stiffness and stress relaxation of the hydrogel. Alkaline phosphatase staining is shown in blue and indicates osteogenic differentiation. Panel reprinted by permission from Macmillan Publishers Ltd: Nature Materials, ref. 200, copyright 2015. (top right) Patterning of vitronectin guides differentiation of MSCs in PEG hydrogels; regions of vitronectin are indicated by white boxes. Osteocalcin staining, indicative of osteogenic differentiation, is shown in green, and cell tracker dye is shown in red. Panel reprinted by permission from Macmillan Publishers Ltd: Nature Materials, ref. 238, copyright 2015. (bottom left) Lower fiber stiffness promotes spreading of MSCs on fibrillar dextran based hydrogels. Cell area is indicated in yellow. Panel reprinted by permission from Macmillan Publishers Ltd: Nature Materials, ref. 247, copyright 2015. Scale bars are 50 μm. (bottom right) Photodegradation of channels within PEG hydrogel enables encapsulated fibrosarcoma cells to migrate through 3D channels. PEG hydrogel indicated in red. Panel from ref. 233. Reprinted with permission from AAAS. It should be noted that there can be coupling between these different properties of the gel.

Development of engineered biomaterials for cell culture has driven key advances in our understanding of cell–ECM mechanotransduction. As described earlier, 2D collagen-coated polyacrylamide gels have long served as a robust platform to investigate the role of stiffness on impacting various cell behaviors.^148^ For example, with this material system, it has been found that substrate stiffness impacts cell migration,^149^ neuronal cell branching,^150^ cell spreading,^151^,^152^ myotube differentiation,^153^ the phenotype of mammary epithelial cells,^12^ and differentiation of MSCs.^11^ However, a number of studies have demonstrated that cell adhesions, signaling, and downstream behaviors, are impacted by culture dimensionality, and pointed to 3D culture models as being more relevant for various physiological contexts and biological processes.^154^–^158^ These and other studies have identified degradability, mechanics, ligand density, and pore size as key characteristics of biomaterials that cells may respond to (Fig. 3). Historically, 3D culture studies were first performed using reconstituted hydrogels of native ECM proteins such as fibrin, collagen, and basement membrane proteins derived from the Engelbreth–Holm–Swarm (EHS) mouse tumor (most commonly using commercial product matrigel).^158^–^160^ However, these materials are generally soft, with elastic moduli typically ranging from 100 Pa–1 kPa,^161^–^163^ and offer limited control over physical properties. This has motivated the development of various engineered biomaterial systems for 3D culture, and the use of these biomaterials to elucidate the impact of mechanical cues on cells. In this section, we will review commonly used engineered biomaterials for 3D culture, cover some recent trends in biomaterial design and development, and describe some of the insights into cell–ECM mechanotransduction gleaned from these new innovations. We further refer the reader to a number of excellent reviews published recently in this space.^164^–^168^

### Commonly used engineered biomaterials for 3D culture

Poly(ethylene glycol) or PEG, alginate, hyaluronic acid (HA), and peptide hydrogels are widely used as engineered biomaterials for 3D culture. Of these, the most commonly used materials are PEG-based hydrogels. PEG is a synthetic polymer that is hydrophilic, presents no binding sites for cells, exhibits minimal protein adsorption, is not degradable by mammalian enzymes, and can be commercially obtained with various lengths, geometries, and functionalized end groups.^169^,^170^ PEG molecules can be crosslinked into 3D hydrogels through various chemistries. Common chemistries include radical photo-polymerization,^171^ Michael addition,^172^ or enzymatic crosslinking,^173^,^174^ depending on the functionalization of the PEG. While PEG is inert to cells, cell adhesion to PEG hydrogels can be facilitated and controlled through coupling of short peptide binding motifs such as RGD to the hydrogel.^175^ PEG hydrogels are nanoporous, and biological processes such as cell spreading, proliferation, and migration are inhibited sterically unless the hydrogels are engineered to degrade.^176^–^178^ Degradability is engineered through introduction of matrix metallo-proteinase (MMP) degradable crosslinks^178^–^180^ or hydrolytically degradable sections into the polymer network.^176^ Stiffness is tuned by varying crosslinking density or polymer concentration from 100s of Pa to above 100 kPa. Some select findings from studies with such PEG-based materials in 3D culture are that altered stiffness regulates smooth muscle cell phenotype,^181^ enhanced stiffness in the absence of RGD promotes osteogenic differentiation of MSCs,^182^ and identification of an optimal stiffness and degradability for intestinal stem cell expansion and organoid formation.^183^

In addition to synthetic PEG hydrogels, hydrogels from the natural biopolymer alginate serve as another common biomaterial system used for 3D culture. Alginate is a copolymer containing guluronic acid (G) and mannuronic acid subunits that is derived from algae.^184^,^185^ Alginate is not degradable by mammalian enzymes and can be crosslinked into a 3D hydrogel ionically, with divalent cations such as calcium, or covalently.^186^ Like PEG, alginate does not present any binding sites for cell adhesion receptors, but RGD can be covalently coupled to alginate to promote cell adhesion.^185^ Interestingly, increased ionic crosslinking leads to enhanced stiffness, tunable over the range of ∼1 kPa to ∼100 kPa, but does not impact pore size.^187^ This is due to the zonal nature of alginate crosslinking, whereby ionic crosslinks connect regions on the alginate polymer that consist of a series of G residues or G-blocks. Additional crosslinkers fill in the crosslinking zone vacancies once the G-blocks are aligned, strengthening the crosslinks but not changing the pore size. A study of MSC differentiation in RGD-coupled alginate hydrogels found an optimal stiffness for osteogenic differentiation from 11–30 kPa, and associated this with optimal binding and clustering of RGD ligands.^187^

In contrast to alginate and PEG, HA is found naturally in various tissues, and cells can bind to HA directly through the cell surface proteins CD44 and RHAMM.^188^,^189^ As with PEG, various schemes have been employed to crosslink HA polymers into 3D hydrogels, stiffness can be tuned from ∼1 kPa to ∼100 kPa by tuning crosslink density or polymer concentration, RGD ligands can be coupled to the HA to promote integrin-based adhesions, and degradability can be modulated by making the crosslinks degradable by MMPs.^190^–^194^ Altered stiffness in covalently crosslinked and non-degradable HA hydrogels did not impact MSC differentiation, with adipogenic differentiation in all cases, while degradation in HA hydrogels promoted spreading, localization of the YAP transcriptional regulator to the nucleus, and osteogenic differentiation.^193^,^195^ Finally, self-assembling polypeptides form a class of hydrogels that are being increasingly used for 3D culture. However, as these materials have been less commonly used for studies of mechanotransduction to date, the reader is referred to a number of excellent reviews for more details on these materials.^196^–^199^ Each of these material systems affords varying degree of control over stiffness, degradability, and ligand density.

### Viscoelasticity and nonlinear elasticity

While work examining the impact of ECM mechanics on cells has focused almost exclusively on the effect of altered elasticity (or stiffness, as used in this review), biological tissues and ECM are often viscoelastic and exhibit nonlinear elasticity.^20^ Viscoelastic materials display some properties of elastic solids and viscous liquids, and as a result, exhibit a time dependent response to a strain or stress. For example, in response to a constant strain, or deformation, viscoelastic materials will exhibit an initial stress, corresponding to an initial resistance to deformation, but this stress will then be relaxed over time to varying extents. Alternatively, in response to a constant stress, or load, viscoelastic materials will exhibit an initial elastic deformation, followed by creep of the material over time. Various soft tissues, such as adipose, brain, breast, bone marrow, and liver, and reconstituted ECMs, consisting of collagen, fibrin, or reconsituted basement membrane (rBM) matrix, are naturally viscoelastic and exhibit substantial stress relaxation or creep.^20^,^200^–^202^ ECM viscoelasticity has various molecular origins including movement of water in the matrix and unbinding of weak crosslinks followed by matrix flow, all of which dissipate elastic energy. Some viscoelastic materials, including reconstituted ECMs and some tissues, are also viscoplastic and exhibit permanent deformations in response to stress or strain.^202^ In addition to being viscoelastic, tissues and ECMs can be nonlinearly elastic or have a strain-dependent elasticity. Nonlinear elasticity in tissues and biopolymer materials often takes the form of strain stiffening, or an increase in resistance to deformation at higher strains.^162^ Interestingly, viscoelastic and nonlinear elastic properties are coupled in fibrin and collagen gels, which both exhibit strain enhanced stress relaxation.^201^ For cells pulling on viscoelastic or nonlinear elastic matrices, the resistance to this pulling becomes a complex function of time and strain. As cells are thought to sense elasticity by gauging resistance to traction forces,^1^,^2^,^12^ viscoelasticity and nonlinear elasticity would be expected to cause time and strain dependent resistance to traction forces and thereby mediate cell–ECM mechanotransduction. This section will cover the development of biomaterials engineered with tunable viscoelasticity or nonlinear elasticity and recent findings that have shown that ECM viscoelasticity and nonlinear elasticity strongly regulate cell biology.

The impact of viscoelasticity on cell behaviors has been demonstrated in 2D culture. Cooper-White and colleagues modulated the loss, or viscous, modulus of acrylamide gels independent of the initial elastic modulus, or stiffness, by varying both the polymer and crosslinking concentration.^203^ When culturing human MSCs (hMSCs) on these substrates, they found that an increased loss modulus promoted cell spreading and stress fiber formation. In another 2D culture study, U2OS osteosarcoma cells and 3T3 fibroblasts were cultured on ionically or covalently crosslinked RGD-coupled alginate hydrogels, with ionically crosslinked alginate being viscoelastic and exhibiting stress relaxation while covalently crosslinked alginate being primarily elastic.^204^ When plated on substrates with a low initial elastic modulus and high ligand densities, cells spread, formed stress fibers, and exhibited higher levels of proliferation and YAP activation only on substrates with stress relaxation. Surprisingly, in both studies, cells on the more viscous or stress relaxing substrates behaved as though they were on stiffer elastic substrates, indicating that cells are not simply integrating the elastic modulus over time when sensing substrate mechanics.

More recently, various approaches have been used to engineer biomaterials with tunable stress relaxation or nonlinear elasticity for 3D culture. In RGD-coupled PEG hydrogels, the use of reversible dynamic bonds instead of stable covalent crosslinks resulted in hydrogels that exhibited stress relaxation.^205^ Varying the stoichiometric ratio of two such reversible bond chemistries allowed control over the timescale of stress relaxation independent of the initial elastic modulus. When cultured in hydrogels with faster stress relaxation, myoblasts were able to spread to a greater extent,^205^ and embryonic stem cell-derived motor neurons were able to form neurite outgrowths.^206^ A different approach was taken to modulate stress relaxation in ionically crosslinked alginate hydrogels.^200^ The stress relaxation rate of viscoelastic alginate gels was enhanced through both reducing alginate polymer length, while holding overall alginate polymer concentration constant, and using PEG spacers to distance the crosslinking junctions. The initial elastic modulus in the hydrogels was maintained by modulating concentration of ionic crosslinking. In RGD-coupled alginate hydrogels, it was found that faster stress relaxation promoted spreading and proliferation of 3T3 fibroblasts. Interestingly, both the initial elastic modulus and the rate of stress relaxation regulated differentiation of MSCs, with faster relaxation promoting osteogenic differentiation of MSCs and formation of an interconnected bone-like matrix in hydrogels with an initial elastic modulus of 17 kPa. This is in contrast with the findings from covalently cross-linked RGD-coupled HA hydrogels,^193^ suggesting cells are sensitive to the difference between the very slow relaxing viscoelastic hydrogels and covalently crosslinked elastic hydrogels. In addition to these alginate and PEG-based approaches, an approach for modulating viscoelasticity in peptide hydrogels was recently reported.^207^ While less work has been done in engineering biomaterials with tunable nonlinear elasticity, a recent study introduced an approach to modulate stress stiffening in polyisocyanopeptide-based hydrogels.^208^ It was found that stress stiffening in soft hydrogels promoted osteogenic differentiation of MSCs.^208^ These advances in mimicking nonlinear elasticity and viscoelasticity of biological tissues will help elucidate the role of complex mechanical properties in regulating biological processes.

### Towards physiologically relevant presentation of ligands

While many of the studies described above use RGD to promote cell adhesion, an emerging body of work has focused on incorporating full-length proteins into engineered biomaterials for 3D culture. The motivation for this is multifold. RGD is a cell adhesion peptide motif found in fibronectin, vitronectin, and other ECM proteins.^209^,^210^ However, signaling from the RGD cell adhesion peptide motif may not replicate the full biological signaling of any of the full-length proteins.^211^,^212^ For example, fibronectin contains a synergy sequence that enhances cell adhesion,^213^ and forces open up cryptic binding sites in fibronectin that further modify its biological activity.^214^,^215^ In addition, consensus cell adhesion peptide motifs that mimic the key signaling behaviors from other cell adhesion proteins such as laminin have not been found. Further, other non-integrin binding proteins, such as growth factors or cell adhesion proteins, may play a potent role in mediating cellular behaviors.^216^ For a more physiological presentation of ligands, ECM or other proteins can be directly tethered to a synthetic hydrogel network.^217^ Using this approach to couple ECM proteins – fibronectin, laminin, collagen IV, or vitronectin – and cell–cell contact proteins – epithelial cell adhesion molecule (EpCAM), E-cadherin, FN9-10 – to PEG, it was found that laminin and EpCAM improved the generation of induced pluripotent stem cells.^183^ Another approach to presenting physiologically relevant ECM proteins to cells in 3D hydrogels involves the formation of interpenetrating networks (IPNs) of a biomaterial with an ECM protein network. In this approach, the biomaterial network is used as a handle to tune the mechanical properties of the networks, while the protein network provides biologically relevant signaling. IPNs of collagen and agarose have been formed for culture of glioma cells.^218^ Increased stiffness, due to an increase in agarose concentration, inhibited invasion of the glioma cells by providing a steric barrier to motility.^218^ Similarly, in IPNs of PEG and rBM matrix, increased stiffness of the matrices limited growth of breast cancer cells.^219^ Both approaches utilized increased density of the inert non-ECM network to enhance stiffness. In contrast, the stiffness of alginate–rBM IPNs was increased by using greater concentrations of calcium crosslinker concentration, and modulation of stiffness did not substantially alter pore size or ligand accessibility.^161^ In soft alginate–rBM IPNs, MCF10A cells, often used to model normal mammary epithelium, formed organotypic acinar structures.^220^ Enhanced stiffness of rBM–alginate IPNs promoted a malignant phenotype marked by enhanced proliferation and invasiveness, and this phenotype was mediated through inhibition of β4 integrin clustering and hemidesmosome formation, and activation of Rac and the PI3K pathway. In contrast, malignant phenotypes of MCF10A cells induced by increased stiffness of acrylamide gels in 2D culture were mediated through β1 integrin clustering, activation of Rho, FAK, and ERK.^12^ This contrast highlights the difference in biological behaviors induced by different ECM model systems. IPNs of alginate with collagen and fibrin have also been used for 3D culture studies.^221^,^222^ A similar IPN-like approach could potentially be taken using de-cellularized ECMs harvested from tissues.^223^,^224^ While more challenging to include than cell adhesion peptide motifs, use of full-length proteins in engineered 3D culture matrices may be necessary for creating robust biomimetic ECM to support specific biological processes.

### Bio-orthogonal crosslinking

Over the last decade, various new crosslinking chemistries have been developed that are bio-orthogonal. This is motivated by the fact that many commonly used crosslinking chemistries have off-target impacts on cells. For example, Michael addition or radical photopolymerization of PEG hydrogels can also crosslink thiols on surface receptor proteins directly to the PEG.^225^ Alternatively, UV light-mediated photopolymerization can damage DNA in cells and generation of radicals can damage cells.^226^,^227^ To avoid such off-target effects, various bio-orthogonal chemistries for crosslinking of hydrogel materials have been explored, and click chemistries have emerged as ideal for bio-orthogonal crosslinking. Click chemistries are a family of crosslinking reactions between molecular pairs that are highly selective and proceed with high efficiency.^228^ Anseth and colleagues introduced the use of click reactions for crosslinking of a PEG hydrogel, and demonstrated the compatibility of these reactions with 3D cell culture.^229^ Similar chemistries have since been adapted for crosslinking in hydrogels of HA, alginate, and gelatin.^230^–^232^ Bio-orthogonal crosslinking approaches are likely to be increasingly adopted in engineered biomaterials.

### Dynamic control over engineered matrices

There has been a major effort towards development of dynamic control over the mechanical and biological properties of engineered matrices recently. This effort was motivated in part by desires to mimic various stiffening and softening observed physiologically in some tissues and diseases as well as spatial heterogeneity within tissues, and to better understand the time dependence of mechanotransduction. Anseth and colleagues engineered photodegradable PEG hydrogels.^233^ Use of a focused laser enabled spatiotemporal control over the hydrogel and RGD degradation, and controlling irradiation intensity and exposure allowed precise control over the degree of gel softening. This approach was used to demonstrate that hMSCs can exhibit mechanical memory when cultured on stiff gels for a sufficient amount of time, as evidenced by sustained nuclear localization of YAP and RUNX2 when the materials were softened.^234^ Conversely, Michael addition mediated crosslinking followed by photocrosslinking of HA gels allows temporal control over gel stiffening.^235^ Induction of osteogenic differentiation of MSCs cultured initially on soft 2D substrates depended on when the gels were stiffened.^235^ In 3D culture, osteogenic differentiation of MSCs in degradable HA gels was inhibited if the gels were covalently crosslinked after the MSCs were allowed to spread, demonstrating a decoupling of cell shape from differentiation in 3D culture.^193^ Other techniques to control photo-activation,^236^ irreversible photo-tethering,^237^ and photo-reversible tethering^238^ of cell adhesion ligands or proteins to PEG hydrogels have also been established. Ionically crosslinked alginate-based hydrogels can be softened and stiffened in bulk through agents that chelate calcium or addition of calcium.^239^ Local control over alginate stiffening and softening was recently achieved through light triggered local release of chelating agents or calcium from liposomes within the gel.^240^ Using this approach to control stiffening of rBM-alginate IPNs, the impact of enhanced stiffness on MCF10A cells was determined after the cells had been cultured in soft IPNs and had formed organotypic acini.^241^ It was found that enhanced stiffness induced malignant phenotypes in the acini. Since these acini were each surrounded by a layer of secreted basement membrane, this finding suggests that mammary epithelium may sense mechanics beyond the basement membrane and into the stromal tissue. As with this approach, dynamically tunable matrices can be used to better model various physiological processes, providing more physiologically relevant contexts to understand cell–ECM mechanotransduction.

### Fibrillar hydrogels

While PEG, HA, and alginate hydrogels are typically nanoporous, many biological tissues and ECMs are microporous and often contain fibrillar ECM proteins such as collagen, fibronectin, or fibrin. The nano-porosity of non-degradable hydrogels can serve as a steric barrier to cell spreading, cell division, and other morphological changes in the absence of hydrogel degradation.^176^,^178^,^179^,^193^,^200^ Further, at the nanoscale, protein fibers themselves can display an elastic modulus on the order of tens to hundreds of MPa,^242^,^243^ and cells may, in principle, sense fibrillar mechanics at the nanoscale, in addition to the nanoscale topography. Indeed, changing the attachment of collagen on a surface from gold-nanoparticles spaced 60 nm apart to nanoparticles spaced 190 nm apart diminished spreading and promoted differentiation of keratinocytes.^244^ One approach to forming fibrillar materials for cell culture has involved the use of electrospinning to form nano or microfibers of engineered materials.^245^ Recent work showed that MSCs on polycaprolactone (PCL) electrospun nanofibers displayed shape changes, cytoskeletal reorganization, genetic regulation, and bone differentiation that are extremely similar to MSCs cultured on flat PCL scaffolds with differentiating media.^246^ Another study used electrospinning to decouple stiffness at the nanoscale relative to bulk stiffness in synthetic fibrillar hydrogels that are microporous. Chen and colleagues developed a dextran fiber network in which the authors could tune the diameter, density, and alignment of the RGD-containing dextran fibers making up the network and thereby tune the elasticity of the fibers between 100 MPa and 3 GPa while tuning the bulk network elasticity from 1 to 50 kPa.^247^ Interestingly, cells spread to a greater extent on substrate with lower fiber stiffness, as lower fiber stiffness facilitated cell clustering of fibers. This contrasts with the common finding that cells spread to a greater extent on stiffer acrylamide hydrogels.^151^,^152^ While these studies were performed in 2D, a combined fibrillar and 3D hydrogel culture approach using a photo-crosslinkable PEG microribbon-based hydrogel scaffold was recently developed, in which biochemical, mechanical, and topographical properties could be independently controlled.^248^ These and other innovations may help elucidate the role of local *versus* global mechanics and nanotopography in cell mechanotransduction. Table 2 summarizes some of the different approaches for designing hydrogels with tunable viscoelasticity, nonlinear elasticity, physiologically relevant ligands, bio-orthogonal crosslinking, dynamic tunability, and fibrillarity.

**Table 2:** Advances in engineered materials for 3D culture that have been used in mechanotransduction studies

	PEG	Alginate	Others
Tunable viscoelasticity	• Reversible bonds used to crosslink PEG	• Change in molecular weight of alginate and PEG spacers • Covalent *vs.* ionic crosslinking of alginate	• Physically crosslinked peptide based hydrogels

Tunable nonlinear elasticity			• Polyisocyanopeptide-based hydrogels

Physiologically relevant ligands	• Tethering of ECM proteins directly to PEG network • Interpenetrating networks of PEG and rBM matrix	• Interpenetrating networks of alginate and rBM matrix, collagen, or fibrin	

Bio-orthogonal crosslinking	• Click chemistry to crosslink PEG	• Click-alginate	• Click-hyaluronic acid-based hydrogels • Click-gelatin

Dynamically tunable gels	• Photodegradable crosslinks to soften gels • Photoreversible tethering of ECM proteins to PEG	• Stiffening or softening through phototunable release of calcium crosslinker or chelator	• Photocrosslinking of hyaluronic acid based hydrogels

Fibrillar hydrogels	• PEG-based microribbons		• Electrospun dextran (2D)

## Advances in imaging technology

In the last 10–15 years, the imaging world has seen a renaissance of new, or in some cases old, technology with substantially improved performance and features. This has expanded the tool box for morphological and molecular imaging well beyond the classical state-of-the-art. Concurrently, microscopy tools have assumed a larger role in cell biology, as localization and morphology, and not just total amount, of intracellular molecules are recognized to be important in mediating cell behaviors. For example, cytoskeletal organization,^1^ transcription factor localization,^41^ and even chromatin organization,^14^ are key indicators of cellular phenotype. The (now well-documented) effects of global ECM substrate stiffness on cell shape and organization of the actin cytoskeleton in mesenchymal cells^249^ were breakthrough discoveries only possible with effective microscopy tools. The same can be said for experiments showing how transcription factors relocalize – but do not change in expression level – in response to intracellular tension developed in MSCs cultured on different stiffness (2D) ECM substrates. Finally, the demonstration of changes in chromatin architecture in response to (2D) ECM substrate stiffness reinforces the notion that multiscale organization: from multiple cells in tissues to intracellular organelles (including the cytoskeleton), and DNA all reflect a biological “state” in response to ECM mechanical cues. Importantly, because of the knowledge gleaned from visualizing cellular and intracellular organization (as dependent variables), clever experiments have shown that controlling cellular architecture or cytoskeletal morphology can in turn direct cell fate.^36^,^250^

These (and most) seminal breakthroughs to date in cellular mechanotransduction or cell–ECM interaction related to intracellular spatial organization have come from classical 2D cell culture systems. The transition from investigating cell–ECM physiology in 2D experimental systems to true 3D environments has been challenging in many aspects, *e.g.* performing biochemical assays such as measuring secreted trophic factors or cell metabolism (for reasons beyond reduced molecular diffusion in 3D systems) to microscopic visualization of protein and cytoskeletal organization due to substantial blur from out-of-focus features and excessive photobleaching. The wide accessibility of confocal laser scanning microscopy (CLSM) and multiphoton fluorescence microscopy (MFM) have substantially increased the ability of researchers to interrogate intracellular organization and cell shape in 3D environments as they offer sub-cellular resolution over ∼300 μm depth (for CLSM) and up to ∼1 mm (for MFM).^251^ For example, CLSM has enabled reliable quantification of cell shape on 2D and in 3D ECMs, which has led to a link between cell shape at early times and cell fate in terms of MSC differentiation at later times.^252^,^253^ Similarly, using MFM it is now possible to study cytoskeletal (re)organization in cells and ECM protein deposition by cells at macroscopic depths into cell–ECM systems.^254^ While these imaging methods have become the staple tools for studying cell–ECM interactions in 3D microenvironments, recent developments in advanced microscopy methods offering better resolution, light management, temporal speed, and contrast mechanisms have largely gone unnoticed by the cell–ECM community. As mentioned above, distinct multiscale spatial organization is an emergent property that characterizes (and also controls) cell–ECM interaction and mechanotransduction, so incorporating improvements in imaging technologies is essential to further our understanding of how cells and their ECM reciprocally influence each other. The following sections are certainly not meant to be an exhaustive review of all developments in bioimaging in the last 15 years, but rather serve to highlight newer methods that offer augmented capabilities specifically for studying cell–ECM interactions in 3D environments.

### Nanoscale biological imaging

Until recently, electron microscopy (EM) was the only method capable of achieving true nanoscale (sub 100 nm) spatial resolution in biological samples. While it is, in principle, possible to both identify proteins and organelles with EM alone, fluorescence microscopy is a much easier way to localize proteins within cell or tissue samples. The combination of fluorescence to complement EM broadens the possibilities for protein–organelle localization; however, fluorescence microscopy and EM have generally been performed on independent samples. With the use of fiducial markers for sample alignment and improved sample processing methods, it is now possible to perform correlative electron light microscopy, or CLEM, on the same sample. The side-by-side comparison of EM and fluorescence images has proven powerful for resolving the interaction of specific proteins with particular cellular organelles with nanoscale structural resolution.^255^ Resolving true nanoscale features of both protein organization (fluorescence) and cellular structure (EM) imaging has been empowered by the development of super-resolution fluorescence microscopy wherein the classical diffraction limit, set by the wavelength of light, was surpassed by using single molecule localization microscopy (SMLM)^256^,^257^ and point-spread function engineering techniques.^258^ The ∼20 nm lateral resolution in super-resolution fluorescence is comparable to that observed in typical scanning EM images and has opened the possibility for complete nanoscale imaging with CLEM. Specifically, in the original demonstration of SMLM, the authors showed that proteins associated with the Golgi apparatus using SMLM were indeed found in the Golgi of the same cells in transmission EM images.^256^ Additional examples, *e.g.* showing viral trafficking and docking on microtubules and mitochondrial organization, further show the unique ability of CLEM with SMLM fluorescence to exhibit protein specificity *via* fluorescence, organelle specificity from high-resolution EM, and nanoscale resolution in both protein and organelle structures.^259^ Although these, and the majority of other nanoscale CLEM studies, have focused on 2D (planar cell culture) geometries, recent evidence has shown that high resolution CLEM is possible in 3D using EM tomography and interference-based SMLM.^260^ This combination showed colocalization of endocytic proteins epsin and clathrin on clathrin coated structures in mammalian cells^260^ with sub-30 nm (near molecular scale) resolution in all dimensions. Moving towards other mechanosensitive cellular components, CLEM imaging of the actin cytoskeleton has enjoyed a rich history where its usage has led to critical information about network architecture, specifically in regards to the dendritic network in motile cells^261^ and location of actin binding proteins within the dendritic mesh.^262^ In general, CLEM, with or without super-resolution fluorescence, offers an unparalleled combination because of the multiplexed (molecular and structural) information it provides. Given how transcription factor localization and cell (and organelle) shape are now recognized as strong indicators/modulators of cell phenotype, CLEM has a strong potential to provide highly relevant information in cell–ECM and mechanotransduction studies.

**Fig. 4:**
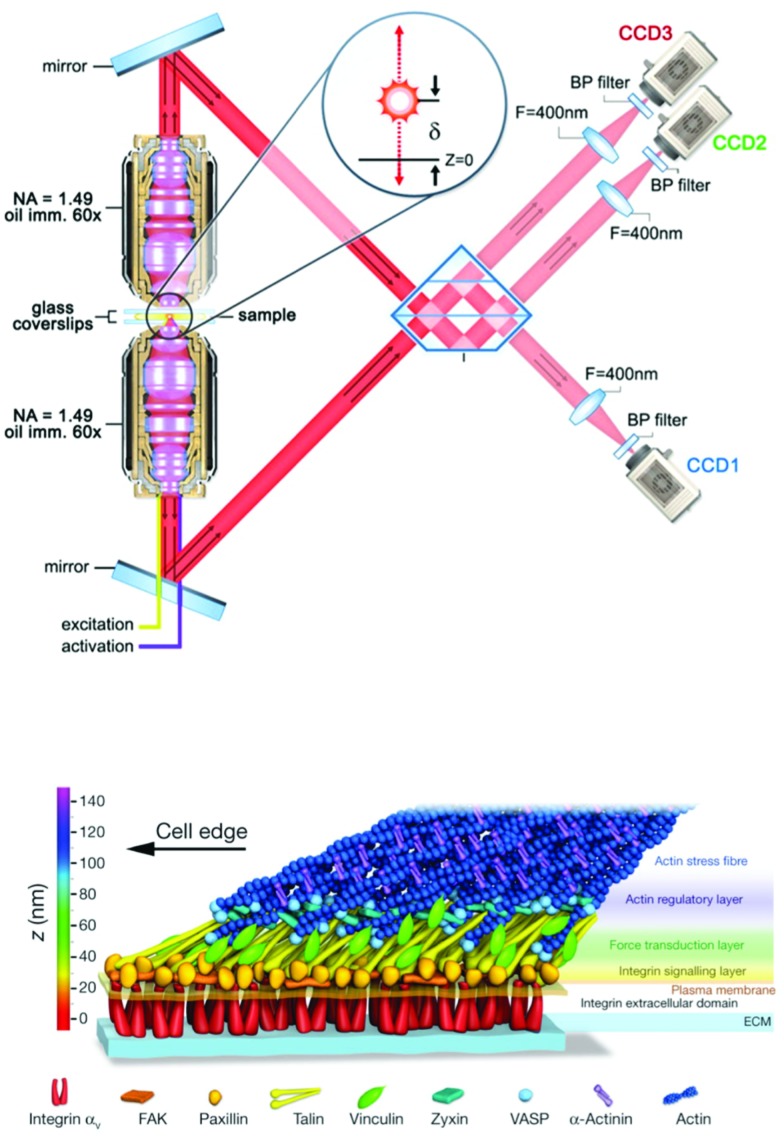
Nanoscale 3D SMLM imaging reveals the molecular architecture of focal adhesion plaques in mammalian cells cultured on glass substrates. (top) Interferometric SMLM provides ∼20 nm resolution in each dimension. Panel adapted from ref. 263. (bottom) Focal adhesion proteins – from cell surface receptors (integrins) to actin stress fibers – are stratified in depth from the substrate in anchored fibroblasts. Panel reprinted by permission from Macmillan Publishers Ltd: Nature, ref. 13, copyright 2010.

Independent of EM, the recent explosion of super-resolution microscopy tools has been making its way into mechanotransduction and cell–ECM studies. Of particular note for the mechanotransduction and cell–ECM communities was a seminal study showing the 3D organization of focal adhesion plaques with 30 nm resolution in all three dimensions using interferometric photoactivated light microscopy – a type of SMLM imaging (Fig. 4).^13^,^263^ This was a demonstrative example showing the power of using newly developed 3D super-resolution microscopy to answer a long-standing question in cell biology: where are proteins located in focal adhesions? While the advantages of both CLEM and super-resolution imaging are clear, two substantial challenges have prevented wide-spread use of both methods in cell–ECM research. First, it is notoriously difficult to produce images from depths beyond one cell height into samples, and second, the required technical expertise in terms of labeling, sample preparation, dye selection, instrument operation, and data processing is significant. Very recent developments in stimulated emission depletion microscopy (STED) have shown ∼60 nm resolution in tissue samples,^264^ and SMLM combined with light sheet microscopy^265^ was shown to permit deep sample imaging while maintaining nanoscale resolution. Such developments have begun to address the first challenge for super-resolution fluorescence imaging whereas tomography is (and has been) the solution for 3D imaging with EM. Tomography is inherently destructive and can only image 10–20 μm deep into a sample. Engineering and technical developments in the coming years are certain to reduce barriers to entry for non-expert users, and potential improvements in 3D performance, especially in the case of fluorescence, are very exciting for those working in the cell–ECM and mechanotransduction fields.

### High resolution, fast imaging in thick samples

The workhorse methodologies for deep tissue imaging have been CLSM and MFM. In both modalities, the sample is “optically sectioned”, meaning that the depth-of-focus is limited such that a sharp image is obtained, even in very deep samples. CLSM permits sharp imaging into samples over ∼300 μm depth by rejecting out-of-focus light with a physical pinhole. MFM provides similar imaging quality over ∼mm depths by virtue of the nonlinear relationship between the excitation light and emitted fluorescence intensities (it is linear for CLSM or epifluorescence), which intrinsically leads to optical sectioning in a sample.^266^,^267^ In practice one achieves an axial resolution ∼500 nm–1 μm with both methods, combined with ∼300 nm lateral resolution. However, both of these methods are traditionally point-to-point (laser scanning) methods, which make them inherently slower than epifluorescence where one acquires an entire frame at once. In addition, light management – in terms of the physical location in the sample from which useful fluorescence is emitted compared to the total volume of sample illuminated – is sub-optimal, especially in CLSM. Recent work has demonstrated three elegant solutions to these drawbacks that stand to substantially increase the usability of these tools in cell–ECM studies: (1) widefield MFM,^268^,^269^ (2) image scanning microscopy,^270^–^273^ and (3) selective plane illumination microscopy (SPIM) or light sheet microscopy.^274^,^275^

MFM, in general, relies on an extremely high photon flux in order to generate nonlinear absorption of light by a dye/protein^276^ and subsequent fluorescence emission. This process is typically achieved using pulsed lasers and is only efficient at or near the focus of an objective lens.^267^ In widefield MFM, the point-to-point process of MFM has been modified to achieve a full image field-of-view (FOV) while maintaining the impressive optical sectioning using a technique called temporal focusing.^268^ This technique elegantly takes advantage of the broad bandwidth of MFM excitation pulses by making the entire bandwidth temporally coincident (*i.e.* compressed) only in the focal plane, thereby reducing the intensity at all other depths in the sample. This is accomplished by dispersing the colors of the pulse with a diffraction grating and re-imaging the grating in the microscope sample plane. This makes it possible to illuminate a large FOV (∼0.5 mm) with a high photon flux instead of only a single diffraction-limited spot. This technique was very recently used to image calcium signaling in mouse cortical columns (0.5 mm × 0.5 mm × 0.5 mm in volume) with sub-cellular cell resolution at 3–6 volumes per second – where each volume contained 43–50 frames. This high-speed imaging allowed for full reconstruction of calcium transients over the many neurons in the hippocampus.^277^ Similar imaging with conventional MFM would take ∼10 minutes per volume, a reduction in speed by at least 100-fold, making it impossible to accurately capture the fast Ca^2+^ transients in the brain. Coupled with the deep penetration of typical MFM excitation lasers (with a wavelength ∼800 nm) and the potential to simultaneously obtain images of additional nonlinear contrast mechanisms (*e.g.* second harmonic generation to image *e.g.* collagen distribution), widefield MFM offers interesting high-speed, high resolution, and deep penetration capabilities for cell–ECM studies.

Image scanning microscopy, or ISM, is a variant of better known structured illumination.^270^,^271^ The uniqueness of ISM lies in its ability to essentially duplicate CLSM performance in widefield (like a spinning disk confocal microscope) with the additional advantage of providing a 2-fold spatial resolution increase, compared to conventional epifluorescence imaging, after computational processing.^270^,^272^ A recent implementation of this technique using all optical signal processing actually provides resolution-enhanced images directly from the microscope and requires only computational deconvolution to obtain the complete image enhancement.^273^ As a potential drop-in replacement for spinning disk confocal microscopy, ISM retains nearly all the characteristics of CLSM: rejection of out-of-focus light, multi-color fluorescence imaging, standard dye compatibility, and the possibility to image deep into 3D samples, with the added benefits of enhanced speed (100s of frames per s) and spatial resolution (∼150 nm lateral/400 nm axial). This method has been used to image the cytoskeleton in red blood cells and fibroblasts in flowing blood and in 3D collagen gels, respectively,^272^,^273^ and its utility in studying cell–ECM interactions will certainly increase with commercialization of the technology by major microscope manufacturers.

**Fig. 5:**
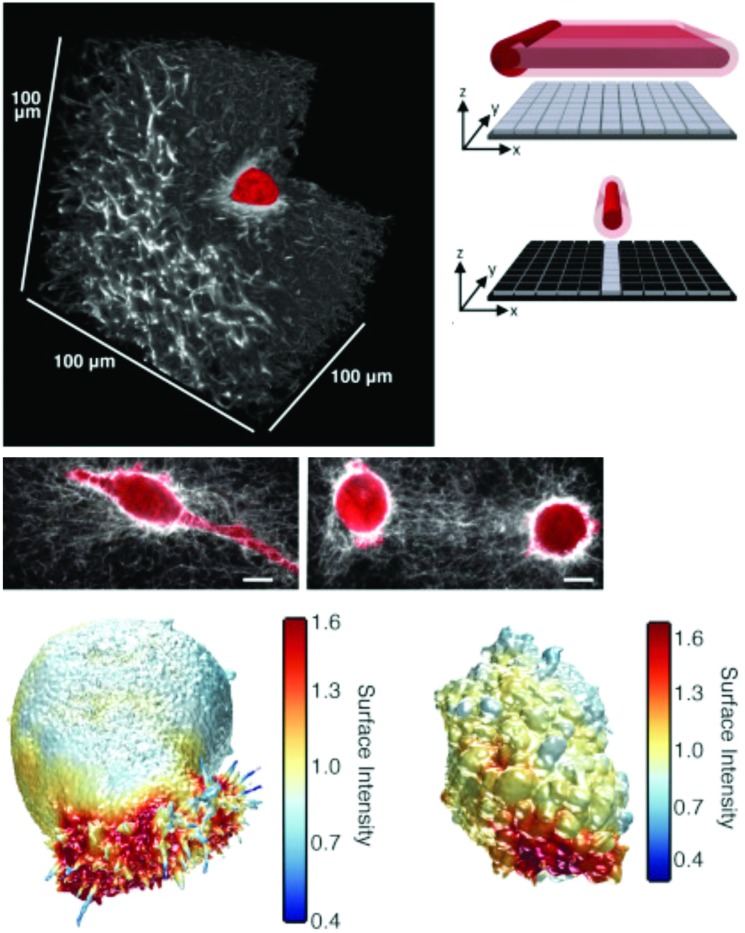
Imaging cell–ECM interaction in 3D collagen ECMs using microenvironmental SPIM (meSPIM). (top right) Principle of meSPIM using line-swept Bessel-beam illumination. (top left) Images of melanoma cells in collagen hydrogel ECMs revealed deformation of collagen and different actin-based protrusions resulting from collagen crosslinking. Scale bar is 10 μm. (bottom) Volumetric renderings showing actin and AktPH local concentration in crosslinked and non-crosslinked collagen gels, left and right respectively. Panels reprinted from ref. 15, copyright 2016, with permission from Elsevier.

SPIM is actually a very old idea,^278^ wherein the excitation is a light sheet (hence the name light sheet microscopy) that illuminates a thin lateral plane of the sample that extends in a direction orthogonal to the imaging objective.^279^ This is extremely effective in terms of light management as only the thin optical section – defined by the lateral extent and thickness of the excitation sheet – from which the image is acquired is exposed to excitation light. In classical CLSM or epifluorescence imaging, the entire sample depth over the desired FOV is exposed to light for every (lateral) 2D frame, meaning that the entire volume of the sample in the FOV is illuminated many times when performing (volumetric) 3D imaging. This unnecessarily produces more phototoxicity and bleaches fluorescent moieties that do not contribute to the unable fluorescent signal for each 2D image in the 3D volume while also potentially adding out-of-focus blur. Imaging spheroids in 3D ECMs^274^,^280^ or deep into living organisms with unparalleled speed,^281^ resolution,^275^,^282^ and duration is possible with SPIM-based methods because of efficient light management – only the portion of the sample that is imaged is actually illuminated. Many derivatives of the basic SPIM setup exist, and one recent study is exemplary in showing how SPIM can be directly used in cell–ECM studies. Welf and colleagues used a nonlinear, Bessel beam SPIM scheme to achieve near isotropic 300 nm resolution at depths more than 100 μm into collagen gels in order to acquire high resolution cellular volumes (∼1 volume per second) and show that melanoma cells in 3D collagen ECMs form actin-driven blebs in uncrosslinked gels but filipodia in crosslinked gels (Fig. 5).^15^ Lattice light sheet microscopy is an alternative development that also uses Bessel beam illumination in the SPIM configuration to image sub-cellular dynamics in 3D for long times.^283^

### Label-free biological imaging

An alternative class of imaging tools that do not require sample labeling with fluorescent molecules is so-called label-free microscopy, a variant of which is the aforementioned second harmonic generation (SHG). These tools have enjoyed their most success in histological applications to determine how sample molecular composition changes in pathologies and in studies of cellular metabolism related to drug delivery or adipogenesis. Because many of these tools arose from physical chemistry and spectroscopy, they provide information on the molecular-scale environment in terms of structure (molecular order and symmetry) in the case of SHG and composition (chemical bonds) in the case of vibrational microscopy. SHG and vibrational microscopy are rapidly growing fields that complement fluorescence microscopy in terms of their information content, and both are readily combined with fluorescence methods. The greatest advantage of label-free methods is the ability to image, track (*e.g.* intracellular organelles), and infer spectroscopic information in biological samples without an exogenous labels. These capabilities prove highly beneficial for long timescale (>24 hour) imaging of, *e.g.* neutral lipids.^284^ There is no dye bleaching, no functional artifact introduced by labeling, and no dye metabolism that complicates interpretation; however, this also means that tracking specific proteins or nucleic acid sequences is difficult, if not impossible, with true label-free methods. This limitation is intrinsic to these methods because nearly all proteins and nucleic acids have the same chemical bonds (composition) in amino acids (and bases), and molecular order (or symmetry) is difficult to assign to specific molecules without *a priori* information. One solution to this challenge is to employ isotopic exchange (*e.g.*, exchange hydrogen for deuterium) in certain amino acids of specific proteins, as has been done in nuclear magnetic resonance for more than 30 years. This introduces a chemical label, albeit with a relatively small size (compared to 25 kDa green fluorescent protein) that is not bleachable.^125^ Nevertheless, the ability to visualize only selected molecular structures with SHG (as explained below) and entire classes of macromolecules, *e.g.* all proteins, DNA, and lipids at once in a sample in vibrational microscopy is advantageous, especially for long time lapse imaging.

SHG microscopy enjoys a growing interest from the cell–ECM community because of its sensitivity to collagen fiber orientation and morphology, due to collagen's non-centrosymmetric molecular architecture. A non-centrosymmetric sample lacks an inversion center (or mirror point) along some spatial dimension, and these materials are called “SHG-active” wherein they can convert two photons of the excitation light into a single photon with exactly half the wavelength. In terms of operating principles and capabilities, SHG exhibits many similarities with MFM and even uses the same excitation source and (point-to-point or widefield) imaging schemes, making it highly attractive as a tool combined with MFM.^285^ Numerous structures including collagen type I,^286^ microtubules,^287^ intermediate filaments,^285^ muscle myosin,^288^ and amyloids^289^ all provide strong SHG signals. SHG intensity and architecture of collagen fibers were recently used as a metric to evaluate aggression of breast cancer cells in 3D matrices.^290^

Vibrational microscopy takes advantage of the intrinsic chemical composition (and structure) of a sample and provides images based directly on molecular vibrations of bonded nuclei, or oscillators, (*e.g.* CH_2_, CH_3_, C=O, or OH vibrations). The most common implementation of this method in biology is *via* Raman imaging, wherein a focused excitation laser is scanned over a sample (like CLSM), and scattered light at different wavelengths can be assigned to molecular vibrations in a sample. By measuring a full vibrational spectrum at each spatial position and scanning over the sample, a hyperspectral dataset is generated. From these spectra, one can integrate specific peaks, corresponding to functional groups in order to create so-called chemical images. A comprehensive review of this technology has been published elsewhere.^291^ Raman cross sections are typically more than seven orders of magnitude weaker than fluorescence cross sections,^292^ making conventional (spontaneous) Raman imaging much slower than fluorescence. Nevertheless, the sensitivity of Raman peaks to, *e.g.* the local environment near these molecular oscillators, leads to characteristic peak shapes and positions. For example, the protein C=O vibration is highly sensitive to protein secondary structure because of unique hydrogen bonding motifs for helices and sheets.^293^–^295^ This ability has been used to visualize transitions in secondary structures of keratin filaments in human hair^296^ and in mechanically loaded fibrin ECMs^297^ with ∼500 nm lateral resolution. Similar to the developments in super-resolution fluorescence microscopy, innovative structured illumination approaches in Raman microscopy have led to an ability to collect Raman spectra with a 2-fold increase in spatial resolution.^298^ Furthermore, the speed challenges in point-to-point imaging of conventional Raman imaging is now being addressed with slit scanning approaches that sacrifice very little in spectral or spatial performance while increasing speed by transferring all spectra from a line at once.^299^ This acquisition method has a side benefit of reducing photodamage because the line illumination can be swept using motorized mirrors to reduce the uninterrupted dwell time of the laser on the sample.^300^

One approach to overcome the small signal generation in Raman imaging is *via* surface-enhanced or tip-enhanced Raman imaging.^301^ While providing single molecule sensitivity, these tools have been confined to 2D geometries or topographical studies and have found limited application in cell–ECM studies. Another method to increase signal throughput compared to spontaneous Raman imaging is by coherent Raman microscopy (CRM), a nonlinear variant of Raman microscopy that increases the signal generation up to six orders of magnitude by active excitation of Raman modes.^302^,^303^ CRM has the added benefit of multiphoton excitation, which restricts the probing volume similarly as in MFM and is readily compatible with 3D imaging. CRM can be operated as a real-time imaging technique by exciting only one Raman mode (with nearly the same instrumentation as MFM and SHG) or in a hyperspectral mode.^304^,^305^ Importantly, because of the rich information content on both intrinsic chemistry and structure of a sample, it has been shown that one can characterize *e.g.* stem cell differentiation by visualizing lipid droplet formation or calcium phosphate deposition directly in unstained, living samples^306^ or different stages of pluripotency in hematopoetic stem cells.^307^ With tangible progress in label-free microscopy technology,^308^ especially in simplifying instrumentation and data processing expertise, and the possible information on molecular composition and structure of ECMs and cells, these tools are likely to find increasing use in future cell–ECM studies. Table 3 summarizes the technical features, challenges, and applications to cell–ECM studies for the imaging technologies described in this section.

**Table 3:** Summary of advances in imaging technology and their features for cell–ECM studies

	Nanoscale imaging	Deep tissue imaging	Label-free imaging
Methods	• Correlated electron light microscopy imaging (CLEM) • Super resolution fluorescence	• Widefield multiphoton fluorescence microscopy • Image scanning microscopy (ISM) • Selective plane illumination microscopy (SPIM)	• Second harmonic generation (SHG) • Vibrational microscopy

Technical features	• Molecular scale spatial resolution in structural features • Molecular scale spatial resolution in protein localization	• High speed, low phototoxicity • High quality imaging in thick (∼100–500 μm) samples	• Molecular composition and structural imaging • Non-discriminant macromolecular localization

Limitations	• Thick samples • Potentially destructive in 3D samples, especially with CLEM • Live-cell imaging	• Data sizes can be extremely large	• Individual protein, DNA, or lipid detection • Relatively low speed • Often requires fixed samples

Applications	• Subcellular organelle stratification • 3D localization of proteins in single cells	• *Ex vivo*, intravital deep tissue imaging • Cell–ECM interaction in 3D hydrogel environments • Morphology in 3D dense tissues	• Cell–ECM interaction in 3D hydrogel environments • Unstained tissue sections

## Outlook

Advances in deciphering cellular mechanotransduction have come from developments in microtechnologies, biomaterials, and advanced imaging. We believe continuous synergy between these three areas will drive new discoveries in cell–ECM interaction and eventually lead to approaches to control mechanotransduction with unprecedented precision. Exciting developments in each of the areas, in addition to deeper applications to cell–ECM studies, are necessary to make the goal of controlling mechanotransduction a reality. More advanced microfluidics and microtechnologies are being developed to provide active mechanical input to single cells, and there is a growing need to investigate cellular mechanotransduction with combined passive and active mechanical inputs, especially in 3D. This is where microtechnology intersects with biomaterials to recapitulate more precise *in vivo* physical microenvironment for *in vitro* studies. Development of 3D biomaterials with controlled mechanics, degradation, and ligand presentation has been a major focus in recent years. The importance of viscoelasticity, as shown in a number of recent works, will likely spark increased development of hydrogel systems based on different natural or synthetic components where viscoelasticity can be finely tuned, perhaps even dynamically. Observing the response of both cells and ECM to dynamic modulation *in situ* will require advanced imaging techniques. Cell–ECM interactions offer a rich biological context for applying state-of-the-art live cell imaging approaches. In particular, observing both ECM and cell dynamics in 3D should reveal critical clues about the nature of cell–ECM interactions. Label-free imaging and spectroscopy approaches offer a complementary approach for monitoring long-term cell–ECM interactions and molecular structure. With so many developments in the fields reviewed here and the ability to combine the different technology areas, it is truly an exciting time to study cell–ECM interaction and mechanotransduction.
